# Incidence of oral cancer in relation to nickel and arsenic concentrations in farm soils of patients' residential areas in Taiwan

**DOI:** 10.1186/1471-2458-10-67

**Published:** 2010-02-12

**Authors:** Che-Chun Su, Yo-Yu Lin, Tsun-Kuo Chang, Chi-Ting Chiang, Jian-An Chung, Yun-Ying Hsu, Ie-Bin Lian

**Affiliations:** 1 Department of Internal Medicine, Changhua Christian Hospital, 135, Nan-Hsiao Street, Changhua 500, Taiwan; 2 Graduate Institute of Statistics and Information Science, National Changhua University of Education, Changhua 500, Taiwan; 3 Department of Bioenvironmental Systems Engineering, National Taiwan University, Taipei, 106 Taiwan

## Abstract

**Background:**

To explore if exposures to specific heavy metals in the environment is a new risk factor of oral cancer, one of the fastest growing malignancies in Taiwan, in addition to the two established risk factors, cigarette smoking and betel quid chewing.

**Methods:**

This is an observational study utilized the age-standardized incidence rates of oral cancer in the 316 townships and precincts of Taiwan, local prevalence rates of cigarette smoking and betel quid chewing, demographic factors, socio-economic conditions, and concentrations in farm soils of the eight kinds of heavy metal. Spatial regression and GIS (Geographic Information System) were used. The registration contained 22,083 patients, who were diagnosed with oral cancer between 1982 and 2002. The concentrations of metal in the soils were retrieved from a nation-wide survey in the 1980s.

**Results:**

The incidence rate of oral cancer is geographically related to the concentrations of arsenic and nickel in the patients' residential areas, with the prevalence of cigarette smoking and betel quid chewing as controlled variables.

**Conclusions:**

Beside the two established risk factors, cigarette smoking and betel quid chewing, arsenic and nickel in farm soils may be new risk factors for oral cancer. These two kinds of metal may involve in the development of oral cancer. Further studies are required to understand the pathways via which metal in the farm soils exerts its effects on human health.

## Background

Heavy metals are trace elements in the environment. Their content and distribution in the air, water or soils are decided by both the nature and human activities. There is already an abundant literature addressing the effects on human health by exposures to these heavy metals [1]. Some of them are known to play a role in the development of human cancers [2]. A notorious example is the increased incidence of lung, skin and bladder cancer caused by exposures to arsenic via drinking well water for residents from the Black Foot Disease endemic area in Taiwan [3,4]. Another example is the increased incidence of cancer in the lungs, nose, and larynx for workers exposed to nickel in the factory [1,5].

Heavy metals in soils may exert their effects on human health via the food grown on them. Vegetable or fruit growing on soils containing a high amount of heavy metals were found to contain higher levels of heavy metals [6,7], too, which may put people eating them under a higher risk of cancer development, if the metal is proved a carcinogen. The distribution of a certain kind of heavy metal in the environment is determined by both the nature and human activities. The advances in human technology may put the latter one a more important and decisive factor [8].

Oral cancer (OC) is one of the fastest growing malignancies in Taiwan [9]. In 1982, its incidence was at 5.12 per 10^5 ^men and 1.54 per 10^5 ^women per year. In 2001, these rates increased to 27.04 and 3.17 per 10^5 ^for men and women, respectively, which were an alarming 5.3-fold increase for men and a two-fold increase for women in a span of only two decades. It is ranked as the fourth most common type of cancer in men, as well as the leading type of cancer that causes death in the same gender between the ages 25 and 44. In contrast, in the United states of America, the United Kingdom, Brazil and many other countries [10-12], the incidence and mortality of OC were either stable or in decline in the past 2 to 4 decades. It is therefore urgent for local scholars to understand the causes behind the opposite trend in Taiwan.

Our previous study [9] showed that the incidence rate of OC in Changhua, at 45.07 per 10^5 ^people per year, was the highest among the 23 counties in Taiwan in 2001. In two decades, from 1982 to 2001, Changhua saw an alarming 6.3-fold increase in the incidence of OC among men. We plotted the incidence against the prevalence of cigarette smoking (CS) and betel quid chewing (BQC), and found that Changhua stood out as the only significant outlier, with an exceptionally high incidence but a mediocre prevalence of BQC and CS. These results suggest to us existence of a new risk factor in this area.

We also found that [13] the age at diagnosis and the prognosis of OC in Changhua were related to the patient's residential area. The mean age at diagnosis was 53.1 and 52.8 for patients living in the north and middle area of Changhua, while that for patients living in the south area was 55.1. Meanwhile, Kaplan-Meier survival curves and log-rank test show patients living in the north and middle area of Changhua have a poorer long-term prognosis. Moreover, the density of electroplating factories is significantly higher in the north and middle areas of Changhua. Since heavy metals are utilized in the industry, and many of these metals are known carcinogens [14,15], it is important to know if exposures to heavy metals are a factor in the development of OC. We also found that OC at Changhua is not limited among factory workers. Most of the OC patients are not working in the electroplating factories. Therefore, a medium via which people can be exposed to heavy metals may be in existence.

Although an abundance of literatures have addressed the carcinogenic effects of various kinds of heavy metal [1], heavy metal and oral cancer has rarely been addressed. The purpose of this study is to explore the spatial relationship between OC and the heavy metal concentrations in soils. Our findings show that the OC incidences in Taiwan are geographically and statistically related to the arsenic and nickel concentrations in farm soils.

## Methods

### Oral cancer registry and the patient's residency

The data from the cancer registration were provided by the Department of Health in Taiwan and contain records of 22,083 patients, who were diagnosed with malignant oral cavity cancer (Appendix) from Jan 1982 to Dec 2002. The residency of these patients is provided in the records of the registry.

### Data on the socioeconomic factors

The data on the personal income [16], factory density 17], factory distribution and types of industry, and other socioeconomic variables were provided by the Financial Data Center, Department of Finance & Statistics, Ministry of Economic Affairs in Taiwan.

### Data on the public behavior toward health

The data were retrieved from a survey conducted in 2002 [18] by the Department of Health. It surveyed the prevalence rates of BQC and CS in randomly selected subjects, including 13,086 men and 12,473 women.

### Data on the soil metal concentrations

The measurement of heavy metal concentrations in the surface (0~15 cm) farm soils were retrieved from a national survey [19], which includes the following 8 metals: arsenic (As), mercury (Hg), cadmium (Cd), chromium (Cr), copper (Cu), nickel (Ni), lead (Pb), and zinc (Zn) [20]. The total concentration of extractable As and Hg in the soil were measured with the aqua regia method, and the concentration of the other six kinds of heavy metals was obtained with the 0.1 N HCl extraction method. By averaging the results of different samples within the same township/precinct, we obtained a value to represent the metal concentration in a certain township/precinct. Except a few alpine areas with very small population and a few metropolitan areas which have very few farms, totally 316 townships/precincts have the measurement of the metal concentrations.

### Data on the Population in Taiwan

**Figure 1:**
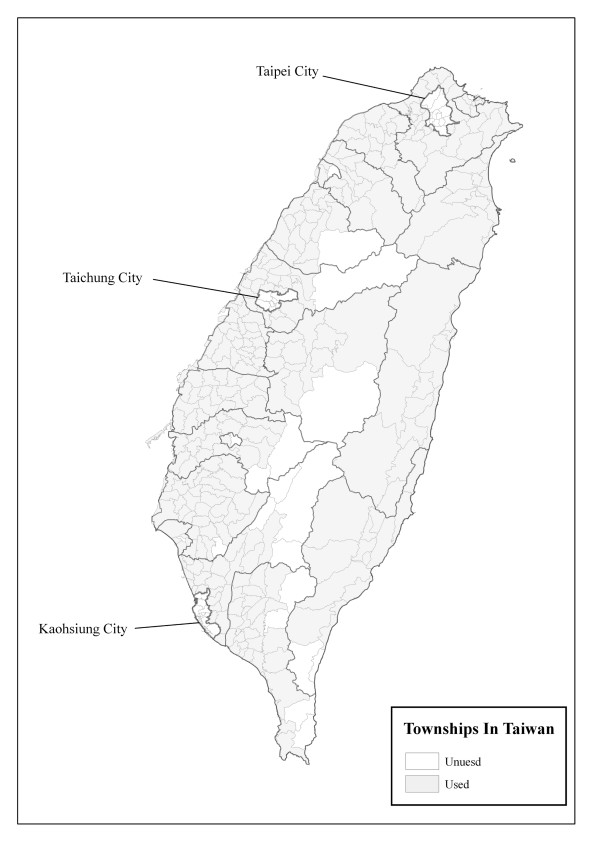
**A map of Taiwan**.

The population in each township/precinct in 2002 is obtained from the Central Institute of Statistics in Taiwan [21]. The population of the 316 townships/precincts in 2002 was 20.4 millions. Analyses were conducted for a subset of 296 townships/precincts, which comprises 83% of the population of the 316 townships (Figure 1). The subset was defined by excluding 3 major metropolitan areas: Taipei City, Taichung City, and Kaohsiung City, which have much higher population density (over 6 thousands people per square mile) than the other cities and more immigrants from other areas. Many precincts in these metropolitan areas have very few farms, and the measurement of metal concentration in farm soil is not available. The inter-quartile size of the population in each of the studied township/precinct range from 19,120 (25^th ^percentile) to 64,540 (75^th ^percentile) people. Each townships/precincts was treated as a unit in the analysis.

### Statistical Methods

**Figure 2:**
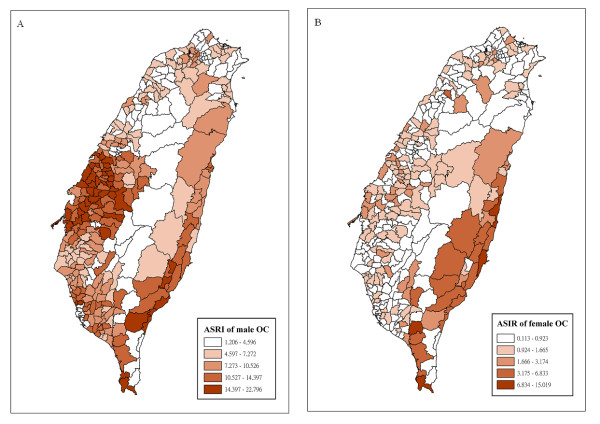
**ASIR (per 10^5 ^people per year) of male OC (A), and female OC (B); prevalence (per 100 people) of BQC (C) and CS (D) in Taiwan**. Of note is that areas with high prevalence of BQC or CS don't correspond well with where the OC incidence is high.

We first calculated the age-standardized incidence rate (ASIR) of OC based on the year 2000 age distribution of the standard population projected by WHO, and showed the incidence for men and for women in Taiwan in figure 2A and 2B. In the dataset on the public behavior toward health, 177 townships/precincts have a sample size more than 50, and subjects from these 177 townships/precincts contributed 97.4% of the questionnaires. We first calculated the prevalence rates of BQC and CS at these 177 townships/precincts, and the rates at the rest townships/precincts were estimated by mean substitution, neighborhood method and indicator Kriging method [22,23]. We found that similar results were obtained with either method. As to the concentrations of the 8 heavy metals in farm soils from the 316 townships/precincts, they were retrievable from the dataset. We then used GIS to map [24,25] the features, and conducted a step-wise regression to explore the associations between ASIR and the 14 explanatory variables (i.e., income, age, density of factory, aboriginal residency areas, prevalence of BQC and CS, and concentrations in the soils for the eight kinds of heavy metal).

The conventional multiple regression was applied at first, and Moran's I [26]. statistics and residual plot were used to examine the spatial autocorrelation of the residuals. If the assumptions of conventional regression are violated, spatial regression is then applied. The spatial models including Conditional Autoregressive Model (CAR) and spatial simultaneous autoregressive (SAR) model [26] were used in the analysis. The neighbor objects in spatial regression were created by distance based method, using centroid to centroid definition with a radius of 10 kilometers to construct spatial proximity matrix. We noted that to use a radius of 20 km or 5 km will result in unrealistic number of neighbors for each object. The analyses were performed using software S-plus with spatial module.

After the important explanatory variables were identified, each of them was stratified into 3 levels, with each level containing roughly one third of the population. We then used histograms to explore the impact of each variable, and the interactions between them. The secular trends of the incidence at different levels of the explanatory variables were also investigated.

**Figure 3:**
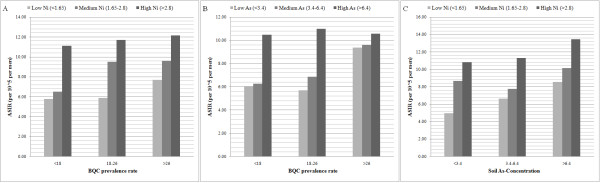
**Incidence of male OC in relation to levels of soil nickel (mg/kg) (A), or soil arsenic (mg/kg) (B), as stratified by levels of BQC prevalence (per 100 men)**. In (C), incidence of male OC in relation to soil nickel concentration, stratified by levels of soil arsenic. Both nickel and arsenic have effects on the incidence of OC.

**Figure 4:**
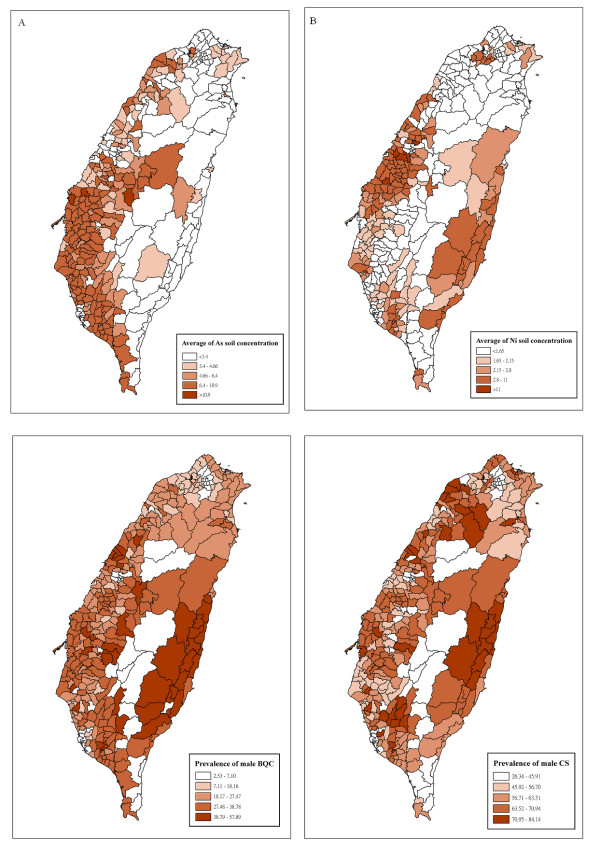
**Concentrations (mg/kg) of arsenic (A) and nickel (B) in farm soils**. Areas with high arsenic are widespread on the west coast. Areas with high nickel are limited to two counties, Changhua on the west, and Hualian on the east coast.

In figure 3A we used the levels 3.4 and 6.4 (mg/kg), which divided the townships into 3 groups with each group containing roughly 1/3 of the population. In figure 4A, we used two more cut points, 4.7 and 10.9 mg/kg, to further divide the middle and high concentration groups, where 4.7 is the medium of the middle group, and 10.9 was used as the cutoff level by the Environment Protection Administration (EPA) of Taiwan to classify the farms with a significantly higher arsenic concentration in soils. Similarly, we used (1.65, 2.15, 2.8, 11) as the cut-points of nickel concentration in figure 4B, and (1.65, 2.8) as the cut-points for figure 3B. EPA used 11 mg/kg as the cutoff value to classify the farms with high nickel concentration.

## Results

### Incidence of OC only partially correspondent to prevalence of BQC and CS

As has shown in figure 2. OC is a male-dominant disease in Taiwan, with male patients constituting 88% [9], we focused on male OC in later analysis, and will give explanations to the male dominance in the discussion. Areas with high incidence are located in mid-Taiwan, along the west coast, where the Changhua County is located (Figure 1). The prevalence of BQC and CS are shown in figure 4C and 4D. Areas with high prevalence of BQC are located along the east coast, which is the main habitat of the aboriginal people, among whom BQC is a tradition and a social rite. For CS, areas with high prevalence are also located along the east coast. A high association between BQC and CS is an observed and documented phenomenon in Taiwan [27]. Based on these maps, it is discernible that the areas with high incidence of OC only partially correspond to areas with high prevalence of BQC or CS.

### Arsenic and nickel in farm soils are related to oral cancer in spatial analysis

Because of strong spatial autocorrelation in the residuals of regular regressions, we used the spatial linear regression to relate the incidence of OC at each township/precinct to the heavy metal concentrations, using variables like age, income, density of factory, aboriginal residency areas, prevalence of BQC and CS as possible confounders.

The conventional multiple regression was applied at first, and the test based Moran's I (0.407) with p-value < 0.001 suggests the existence of spatial autocorrelation of the residuals. The CAR and SAR spatial regression were then applied. Both methods come out with very similar estimation results and have small Moran's I's with p-values > 0.05, though CAR has smaller Moran's I than the SAR. Table 1 shows the results obtained from CAR method, including the full model with all factors, and the concise model after stepwise variable selection procedure. In both models, nickel and arsenic appeared as the only two metals with a statistical significance. Results are similar under different imputation methods for the prevalence of BQC or CS. The following concise model was obtained under neighborhood method:

**Table 1:** Stepwise spatial regression of the male OC incidence at the 296 townships.

		Coef	**95% C.I**.	p-value	mean	SD
		
Full model	const	14.37	(4.99,23.8))	0.003		
	BQC	0.089	(0.03,0.15)	0.003	28.3	9.1
	CS	-0.048	(-0.1,0.01)	0.091	62.4	8.2
	As	0.443	(0.26,0.63)	<0.001	5.42	2.59
	Cd	0.404	(-0.15,0.97)	0.153	0.16	0.77
	Cr	-0.182	(-0.49,0.12)	0.246	1.22	2.53
	Cu	0.078	(-0.04,0.19)	0.178	7.77	8.16
	Hg	-1.892	(-5.76,1.98)	0.339	0.17	0.12
	Pb	-0.058	(-0.18,0.06)	0.347	8.95	4.24
	Ni	0.499	(0.23,0.77)	<0.001	2.76	2.93
	Zn	-0.071	(-0.14,0)	0.054	12.8	12.8
	Income	-0.003	(-0.01,0)	0.288	616	93.8
	Fd	0.004	(-0.01,0.02)	0.620	30.8	32.9
	Age	-0.17	(-0.37,0.03)	0.096	34.8	2.48
	Aborigine	0.702	(-0.72,2.12)	0.334	0.12	0.34
*R^2 ^= 53.52%*						
Moran's I = -0.021		p-value = 0.677				

Concise model	const	3.05	(1.36,4.75)	0.001		
	BQC	0.078	(0.03,0.12)	0.001		
	As	0.466	(0.28,0.65)	<0.001		
	Ni	0.326	(0.17,0.48)	<0.001		
	Aborigine	1.43	(0.14,2.72)	0.031		
*R^2 ^= 50.98%*						
Moran's I = -0.013		p-value = 0.81				

Regression result on prevalence of betel quid chewing (BQC) and cigarette smoking (CS), 8 heavy metal concentrations (As~Zn), average income, age, factory density (Fd) of the area, and indicator for residency of aboriginal (1 if yes, and 0 otherwise). Government defined an area as a residency of aboriginal if more than 40% of its population is comprised of aboriginals. Ni is nickel concentration and As is arsenic concentration.

where *ASIR, BQC, As *and *Ni *and *Aboriginal *stand for the age-standardized incidence rate, prevalence of betel quid chewing, nickel and arsenic concentration (mg/kg) in farm soils, and aboriginal residency (defined at the footnote of Table 1), respectively. The prevalence of CS is not included due to a high correlation with prevalence of BQC.

The spatial regression was applied on the data with various interpolating methods for the BQC/CS prevalence, as well as the data without any interpolating. All came out with similar results that BQC, Ni and As are significantly correlated with the ASIR of OC.

### Arsenic and nickel concentration are associated with the incidence of oral cancer

In figure 4A and 4B, the concentrations of arsenic and nickel in farm soils in Taiwan are shown. Areas with high arsenic concentration in farm soils are widespread over the mid- and southern part of the west coast. For nickel, areas with high concentrations are limited to two areas: Changhua, an agriculture county in the mid-part of the west coast, and Hualien, on the east coast, a mountainous county.

Because BQC is the most important risk factor for OC in Taiwan [27-30], we went on to study the interaction between BQC and arsenic or nickel in farm soils and their effects on the incidence of OC. In figure 3A and 3B, their effects on the incidence of male OC in the non-metropolitan areas is shown. Whether an area had a low, medium, or high prevalence of BQC, both nickel and arsenic in farm soils have an effect on the incidence of oral cancer.

In figure 3C, we show the effects of both metals on the OC incidence. A steady increase in the incidence from areas with low concentration to areas with high concentration of arsenic in farm soils is observed. On the other hand, if we focus on the areas with low, medium, or high content of soil arsenic, the concentration of soil nickel still has a marked effect on the incidence of OC, indicating that these two metals may play important roles in the development of OC.

Results from the corresponding three-way ANOVA (not shown here) of incidence rate classified by Ni, As and BQC indicate that all three are significant (p-value < 0.05). No interaction among them reaches the level of significance, indicating that each factor alone is important.

### Secular trends of Incidence correspond to levels of As or Ni concentrations

**Figure 5:**
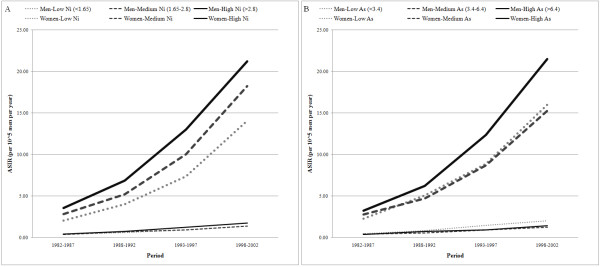
**Secular trend of OC incidence (ASIR) in areas at different levels of Ni (A) and As (B)**. ASIR increases steadily in all areas, regardless of the levels of Ni or As. However, in areas with higher level of Ni, the incidence is higher, too. For As, the levels don't correspond with the OC incidence as well as that for Ni.

The incidences of male OC in the non-metropolitan areas shown in figure 5. A steady increase in the incidence of male OC was observed in the study period, with higher incidences in areas with higher nickel concentrations. As to the effect of arsenic, areas in the upper third level has the highest incidence, but we did not detect marked differences in the incidence between the areas in the mid and lower third levels.

## Discussion

Exposures to hazardous materials in the home, the workplace, and the community can cause or exacerbate a multitude of diseases [31]. Physicians commonly treat the sequelae of these diseases; however, unless we start to recognize the connection between the occurrence of diseases and exposures to hazardous elements, treatment of the manifestations rather than the cause at best only ameliorate the condition. At worst, ignoring the effect of hazardous exposures may both lead to failure of treatment and failure to recognize a public health problem with widespread significance. Nowadays, environmental exposures are increasingly being associated with deterioration in health whose outcomes range from the subclinical to the clinically catastrophic [31].

Despite that many countries have observed a steady or declining OC incidence in the past decades, it is the fastest growing malignancy in Taiwan. We were interested to know that the State of Nevada has also witnessed a trend of increase in the OC incidence, opposite to most other states in the United States of America[10]. It is a surprising coincidence that experts in the University of Nevada, Las Vegas found that there was a severe environmental pollution with heavy metals resulted from widespread and massive landscaping [8].

For the Changhua County in Taiwan, OC is ranked as the third most common type of cancer in men [32]. We found that the prognoses of OC patients diagnosed and treated at the Changhua Christian Hospital[13] are associated with their residencies. Patients who live in areas with higher density of electroplating factories survive shorter, and in areas with lower density, longer.

As stated above, treatment of the manifestations rather than the cause at best can ameliorate the condition. BQC and CS are the established risk factors of oral cancer in Taiwan. A high association between these two habits is also reported [27]. The Government in Taiwan has long recognized the causal relationship between OC and BQC. In fact, a vigorous campaign has been waged to combat OC through public education. People are well informed of the risk accompanying BQC, and chewers are encouraged to abstain from the habit or at least undergo regular check-ups with the dentist for detection of early oral cancer. However, the incidence of OC keeps increasing in recent years.

If we simply look at the prevalence of BQC and CS and the incidence of OC on the map, it is hard to miss the discord. Where the prevalence of BQC and CS is high does not correspond to where the incidence of OC is high. Something may be amiss in the picture of our understanding of the pathogenesis of OC in Taiwan. Our previous studies [9,13] make us suspect that local factors may be present and prompt us to identify the underlying connections with hazardous exposures.

Arsenic is a known carcinogen causing many types of cancer [14]. Hazardous exposures to arsenic present in artesian well water are considered a major cause of the Black Foot Disease (BFD) and other types of cancer on the southwest coast of Taiwan. As has shown in figure 4, concentrations of arsenic in farm soils in areas endemic for BFD are also higher than those in other areas on the island. The government in Taiwan has already completed the construction required to supply the area inflicted with BFD with tap water in the early 1980s. However, despite the disappearance of BFD, the incidence of cancer reported to be associated with hazardous exposures to arsenic remains very high [33,34]. These facts made us wonder if arsenic in farm soils may be playing a role similar to that in artesian well water.

Nickel is also a well-recognized carcinogen, which is known to increase the incidence of nasal cancer and lung cancer among those with hazardous exposures during purification in the factory [35,36]. However, most, if not all authors in the literature[1] described an association between exposures of factory workers and the incidence of a particular type of cancer among them. A widespread effect of this heavy metal on the public in the community, be it small or large, has not been described yet.

Here we present a strong association between concentrations of arsenic and nickel in farm soils, and the incidence of OC. Farm soils can be regarded as either an emitter or a receiver of the environmental toxin. As an emitter, the soil may release toxin to crops, to the groundwater, or to air; as a receiver, it does not matter whether the toxin was initially in the air or water; the residuals eventually settle onto the soil. Therefore the heavy metal concentration in farm soils can fairly reflect the amount of heavy metal present in the environment, and the risk that the residents in the vicinity are exposed to [37]. A question arises then: How do these known carcinogens in farm soils enter the human body? One possible route is via the food grown on them. Vegetable or fruit growing on soils with high contents of heavy metals were found to contain higher levels of heavy metals [6,7], which may put the people eating them under a higher risk of cancer development, as a result. Note that in this study we excluded people living in the 3 major metropolitan areas; people in these areas are more likely to have access to farm products produced far away. To clarify the pathways via which heavy metal in the soils exerts it effects on human health, we are currently teaming up with experts in environmental engineering for further study.

A limitation of this study is that the residency does not necessarily reflect the lifetime exposure, migration may happen. However, the trend here is from the country to the city and the three big cities here are excluded in the analysis.

Another limitation is that the BQC/CS prevalence are available for only 177 townships and the prevalence for the rest townships were obtained by interpolating methods. To verify our results, we tried various interpolating methods as well as the non-interpolating data, and all led to the same conclusion that BQC, Ni and As are significantly associated with OC incidence. In addition to these 3 factors, we also noted the indicator variable of aboriginal residency is significant in some concise models. Areas classified as aboriginal residency have a higher OC incidence rate. However, this variable is strongly confounded with a few variables like age and income, and its effect needs further investigation. Here we focused mainly on the male OC, and the female OC is not addressed. Because only 12% of the 22,083 cases in study are women, the small number will make cases in each township/precinct too few for statistical analyses. On the other hand, a great disparity of BQC prevalence between the genders in Taiwan [38] also makes the estimation of the prevalence of BQC in women difficult. Though BQC is prevalent in men, women rarely practice it. This difference may be related to culture and the image a chewer creates in the public. The results here support our hypothesis: BQC is the inducer, while arsenic and nickel act as promoters in the carcinogenesis of OC. Of course, it is still a hypothesis; more work is needed to prove or disprove it, and we are currently working on it.

## Conclusion

A past study had found an association between the density of electroplating factories and prognosis of oral cancer. This report further extended the finding: beside the two established risk factors, cigarette smoking and betel quid chewing, concentrations of nickel and arsenic in farm soils are also correlated with incidence of oral cancer in Taiwan. We are now studying serum levels of heavy metal in patients and controls to ascertain the evidence of the novel risk factors. Hopefully through the works, more efficient preventive procedures can implemented for public health.

## Competing interests

The authors declare that they have no competing interests.

## Authors' contributions

IBL and CCS conducted the analysis and drafted the manuscript preparation, and editing. CCS provided study concepts, and IBL provided study design through the writing of the manuscript. TKC, IBL, CCS deliberated about definition of intellectual content. IBL, JAC, and CTC tasked acquisition data. YYL assigned statistical analysis during the study. TKC did the manuscript review. All authors read and approved the final manuscript.

## Appendix

I.C.D-O-FT T-140, 141.1-141.9, 143-144,145.0-145.2, 145.6, 145.8, 145.9, 149.8, and 149.9 [9].

## Pre-publication history

The pre-publication history for this paper can be accessed here:

http://www.biomedcentral.com/1471-2458/10/67/prepub

## References

[B1] Navarro Silvera SA Rohan TE Trace elements and cancer risk: a review of the epidemiologic evidence Cancer Causes Control 2007 18 7 27 10.1007/s10552-006-0057-z 17186419

[B2] Boffetta P Nyberg F Contribution of environmental factors to cancer risk British Med Bull 2003 68 71 94 10.1093/bmp/ldg023 14757710

[B3] Wang CH Jeng JS Yip PK Biological gradient between long-term arsenic exposure and carotid atherosclerosis Circulation 2002 105 1804 9 10.1161/01.CIR.0000015862.64816.B2 11956123

[B4] Chen CJ Chuang YC Lin TM Malignant neoplasms among residents of a blackfoot disease-endemic area in Taiwan: high-arsenic artesian well water and cancers Cancer Res 1985 45 5895 99 4053060

[B5] Casdorph H Walker M Toxic Metal Syndrome 1995 Garden City Park, NY: Avery Publishing 150

[B6] LI Y Wang YB Gou X Risk assessment of heavy metals in soils and vegetables around non-ferrous metals mining and smelting sites, Baiyin, China J Environ Sci 2006 18 6 1124 34 10.1016/S1001-0742(06)60050-8 17294953

[B7] Li JT Liao B Lan CY Zinc, nickel and cadmium in carambolas marketed in Guangzhou and Hong Kong, China: Implication for human health Sci Total Environ 2007 388 1-3 405 12 10.1016/j.scitotenv.2007.08.008 17881037

[B8] Mrozek SA Buck BJ Drohan PJ Decorative Landscaping Rock as a Source for Heavy Metal Contamination, Las Vegas, Nevada, S Soil and Sediment Contamination 2006 15 5471 80 10.1080/15320380600847716

[B9] Su CC Yang HF Huang SJ Distinctive features of oral cancer in Changhua county: highest incidence, buccal mucosa preponderance, and a close relation with prevalence of betel quid chewing habit J Formos Med Assoc 2007 106 225 33 10.1016/S0929-6646(09)60244-8 17389167

[B10] Kingsley K O'Malley S Ditmyer M Analysis of oral cancer epidemiology in the US reveals state-specific trends BMC Public Health 2008 8 87 10.1186/1471-2458-8-87 18331638 PMC2287178

[B11] Møller H Brewster D Quinn M, Wood H, Cooper N, Rowan S Lip, mouth and pharynx Cancer Atlas of the United Kingdom and Ireland 1991-2000. ONS SMPS No. 68 2005 Basingstoke: Palgrave MacMillan 129 38

[B12] Boing AF Peres MA Antunes JLF Mortality from oral and pharyngeal cancer in Brazil: trends and regional patterns, 1979-2002 Pan Am J Public Health 2006 20 1 1 8 10.1590/s1020-49892006000700001 17018219

[B13] Su CC Chung JA Hsu YY Age at diagnosis and prognosis of oral cancer in relation to patient's residential area: Experience from a medical center in Taiwan Oral Oncology 2008 44 11 1032 8 10.1016/j.oraloncology.2008.01.015 18620898

[B14] IARC monographs on the evaluation of carcinogenic risks to humans: arsenic and arsenic compounds IARC Monographs 1980 23 397000668

[B15] IARC monographs on the evaluation of carcinogenic risks to humans: nickel and nickel compounds IARC Monographs 1990 49 257 PMC7681426 2232124

[B16] Financial Data Center Department Ministry of Finance, ROC Revenue Statistics in 1999-2002 http://www.fdc.gov.tw/

[B17] MOEA Economic Statistics Database, Department of Statistics, Ministry of Economic Affairs, ROC. Ebook: Manufacturing operation statistical surveys & census, 1999-2000 and 2002 https://2k3dmz2.moea.gov.tw/gwWeb/

[B18] Bureau of Health Promotion, Department of Health, ROC Survey study of public knowledge, attitude and behavior toward health http://olap.bhp.doh.gov.tw/

[B19] Environmental Protection Administration of the Republic of China Survey of heavy metals in the soil samples Statistics Office of Environmental Protection Administration. Yearbook of Environmental Statistics Taiwan Area, the Republic of China. Environmental Protection Administration of the Republic of China, Taipei, 1992, 2002 1989

[B20] Environmental Protection Administration, Executive Yuan, ROC Soil and Groundwater Pollution Remendiation web http://sgw.epa.gov.tw/public/En/index.htm

[B21] Dept of Househlod Registration, Ministry of the Interior, Republic of China Monthly Bulletin of Interior Statistics 2002 http://www.ris.gov.tw/version96/pe_st_reg.html

[B22] Hughes JP Lettenmaier DP Data requirements for Kriging: estimation and network design Water Resour Res 1981 17 6 1641 50 10.1029/WR017i006p01641

[B23] Isaaks EH Srivastava RM Applied geostatistics 1989 New York: Oxford University Press, Inc 259 322

[B24] Vieira V Webster T Aschengrau A A method for spatial analysis of risk in a population-based case-control study Int J Hyg Environ Health 2002 205 115 20 10.1078/1438-4639-00136 12018004

[B25] Lee J Wong DWS Statistical analysis with ArcView GIS 2001 New York: John Wiley and Sons

[B26] Kaluzny SP Vega SC Cardoso TP S+Spatial Stats: user's manual for Windows and UNIX 1997 New York: Springer 226

[B27] Wen CP Tsai SP Cheng TY Uncovering the relation between betel quid chewing and cigarette smoking in Taiwan Tob Control 2005 14 i16 22 10.1136/tc.2004.008003 15923442 PMC1766184

[B28] Ko YC Huang YL Lee CH Betel quid chewing, cigarette smoking and alcohol consumption related to oral cancer in Taiwan J Oral Pathol Med 1995 24 450 3 10.1111/j.1600-0714.1995.tb01132.x 8600280

[B29] Lu CT Yen YY Ho CS A case control study of oral cancer in Changhua County, Taiwan J Oral Pathol Med 1996 25 245 8 10.1111/j.1600-0714.1996.tb01379.x 8835822

[B30] IARC Monographs on the Evaluation of Carcinogenic Risks to Humans Betel-Quid and Areca-Nut Chewing IARC Monographs 1985 37 141 PMC4781453 15635762

[B31] Braunwald E Fauci AS Kasper DL Harrison's Textbook of Internal Medicine 2001 New York: McGraw-Hill 19

[B32] Chen PH Ko YC Yang YH Important prognostic factors of long-term oropharyngeal carcinoma survivors in Taiwan Oral Oncol 2004 40 847 55 10.1016/j.oraloncology.2004.03.006 15288842

[B33] Chiu HF Ho SC Yang CY Lung cancer mortality reduction after installation of tap-water supply system in an arseniasis-endemic area in Southwestern Taiwan Lung Cancer 2004 46 265 70 10.1016/j.lungcan.2004.05.012 15541810

[B34] Yang CY Chiu HF Chang CC Bladder cancer mortality reduction after installation of a tap-water supply system in an arsenious-endemic area in southwestern Taiwan Environmental Research 2005 98 127 32 10.1016/j.envres.2004.07.013 15721893

[B35] Andersen A Engeland A Berge SR Exposure to nickel compounds and smoking in relation to incidence of lung and nasal cancer among nickel refinery workers Occup Environ Med 1996 53 708 13 10.1136/oem.53.10.708 8943837 PMC1128579

[B36] Grimsrud TK Berge SR Haldorsen T Exposure to different forms of nickel and risk of lung cancer Am J Epidemiol 2002 156 1123 32 10.1093/aje/kwf165 12480657

[B37] Boffetta P Epidemiology of environmental and occupational cancer Oncogene 2004 23 6392 403 10.1038/sj.onc.1207715 15322513

[B38] Tsai KY Su CC Lin YY Chung JA Lian IB Quantification of betel quid chewing and cigarette smoking in oral cancer patients Community Dentist Oral Epidemiol 2009 37 6 555 61 10.1111/j.1600-0528.2009.00504.x 19845714

